# Proportion of Brazilian diabetes patients that achieve treatment goals: implications for better quality of care

**DOI:** 10.1186/s13098-015-0107-3

**Published:** 2015-12-14

**Authors:** Deise Regina Baptista, Rubia Daniela Thieme, Walleri Christini Torelli Reis, Roberto Pontarolo, Cassyano Januário Correr

**Affiliations:** Post Graduate Program in Pharmaceutical Sciences, Federal University of Parana, Av. Pref. Lothario Meissner, 632, Jardim Botânico, Curitiba, PR Brazil; CNPq (Conselho Nacional de Desenvolvimento Científico e Tecnológico), Brasilia, Brazil; Program of Pharmaceutical Sciences, Federal University of Parana, Curitiba, Brazil

**Keywords:** Diabetes care, Support network, Goals of good clinical practice

## Abstract

**Background:**

Diabetes and its complications are substantial causes of morbidity and mortality, and caused approximately 5.1 million deaths worldwide in 2013. Early detection and treatment of diabetes complications can prevent their progression.

**Object:**

This study compared the proportions of patients with type 1 and 2 diabetes mellitus (T1DM and T2DM, respectively) who achieved the goals of good clinical control.

**Methods:**

Adults and elderly patients with T1DM and T2DM at a public outpatient endocrinology service in Brazil were retrospectively evaluated between 2012 and 2013. Clinical and socio demographic data were obtained from medical records and evaluated in accordance with the Brazilian Diabetes Society Guidelines. Care process measures, outcomes indicators, and supporting process measures were evaluated.

**Results:**

A total of 1031 records were analyzed: 29 and 71 % of patients had T1DM and T2DM, respectively. T2DM patients had significantly higher BMI than T1DM patients (overweight and obesity in 85.1 vs. 47.5 %, p < 0.01). The follow-up periods for diabetes and number of clinical visits to the endocrinology service were significantly greater among T1DM patients than T2DM patients (p < 0.01). However, T2DM patients required significantly more other (i.e., non-endocrinological) healthcare services (p < 0.01). HbA1c was significantly lower in T2DM patients (p < 0.01). Moreover, blood pressure and triglycerides were significantly higher in T2DM patients (p < 0.01), whereas total cholesterol and low-density lipoprotein were significantly lower in T2DM patients (p < 0.01). Only 0.5 % of the patients achieved all targets, and 1.1 % did not achieve any.

**Conclusions:**

The achievement of goals of good clinical practice varies among the parameters evaluated. Almost no patients achieved all targets. Many patients are overweight and do not achieve targets for HbA1c, lipid profile, or blood pressure control.

## Background

The number of diabetes cases has increased over the last few decades, representing a global epidemic. In 2013, an estimated 382 million people had diabetes, which is projected to increase to 592 million by 2035 [1]. This problem is particularly important when taking into account the clinical, humanitarian, and economic impacts of the condition [2–5]. In the United States of America, diabetes accounts for almost 14 % of healthcare expenditures [6]. Moreover, diabetes is associated with a high prevalence of depression [7] and adversely impacts employment, absenteeism, and work productivity [4].

The disease is classified as type 1 diabetes mellitus (T1DM), accounting for 5–10 % of cases, and type 2 diabetes mellitus (T2DM), accounting for 90–95 % of cases. The cause of T1DM, which often develops during childhood and adolescence, is absolute deficiency of insulin secretion due to a cellular-mediated autoimmune or idiopathic destruction of pancreatic β-cells. Meanwhile, T2DM is characterized by variable degrees of insulin deficiency and resistance, and is commonly present in obese adults and the elderly [1, 8, 9].

Defective insulin secretion and/or action results in hyperglycemia, which is associated with long-term complications, dysfunction, and failure including retinopathy, nephropathy, peripheral neuropathy with a risk of foot ulcers, lower-limb amputations, Charcot joints, autonomic neuropathy, sexual dysfunction, cardiovascular disease, hypertension, and lipoprotein abnormalities [9]. Diabetes and its complications are substantial causes of morbidity and mortality, and caused approximately 5.1 million deaths worldwide in 2013 [1, 8].

Early detection and treatment of diabetes complications can prevent their progression. Eye examinations, urine tests, foot examinations, blood lipid and glucose control, blood pressure and lipid management, medication adherence, smoking cessation, healthy diet, regular physical activity, and maintaining normal body weight are recommended actions. Health outcomes are better when diabetes patients are treated in the context of organized programs with coordinated teams of health professionals [10, 11].

The Support Network for diabetes established by the National Brazilian Health Care System (NBHCS) aims to improve the quality of care for patients with diabetes on the basis of integrality, longitudinality, and health education; ultimately, the program aims to increase treatment efficiency, self-care, and quality of life in an attempt to achieve the goals recommended by guidelines issued by diabetes societies to control cardiovascular risk factors and chronic complications [12, 13]. However, there is a discrepancy between the goals recommended in these guidelines and the values instilled into the patients as well as the frequency of screening for chronic complications; therefore, the quality of care for diabetes patients must be improved [14].

Considering the lack of studies comparing the effectiveness of healthcare for patients with T1DM and T2DM, the present study compared the proportions of patients with T1DM and T2DM who achieved the goals of good clinical practice during routine secondary endocrine care at the NBHCS Support Network.

## Methods

This retrospective cohort study was conducted between January 2012 and December 2013 in a public outpatient clinic at a university hospital in Curitiba, Paraná, Brazil. Adults older than 18 years and elderly people older than 60 years [15] with T1DM and T2DM routinely attending this outpatient endocrinology service were included. This study meets the standards of research in humans and was approved by the Ethics Committee of the Universidade Federal do Paraná, Hospital de Clínicas (#411.484).

Data were obtained from the latest medical records from clinical and physical examinations performed by a physician. Information was limited to medical records involving at least one medical consultation at the outpatient endocrinology service, specifically within the last 6 months. Data were collected on the basis of the goals for adequate metabolic control and management of diabetes recommended by the Brazilian Diabetes Society (BDS) Guidelines [16]. The following variables were assessed: age, sex, period of treatment for diabetes (years), weight (kg), height (cm), body mass index (BMI, kg/m^2^), process measurements, outcomes indicators, and supporting process measurements (not required by the BDS).

Process measurements included the frequency of patients who underwent evaluation of the following parameters at least once in the last 6 months: systolic and diastolic blood pressure (sBP and dBP, respectively, mmHg), glycated hemoglobin (HbA1c) (%), total cholesterol (TC), low-density lipoprotein (LDL) cholesterol, high-density lipoprotein (HDL) cholesterol, triglycerides (TG), abdominal circumference, education for smoking cessation, angiotensin-converting enzyme (ACE) inhibitor or angiotensin II receptor antagonist (ARA2), and aspirin prescription; parameters evaluated in the last year were funduscopy and microalbuminuria upon request, and foot examination.

The outcome indicators were the latest sBP and dBP, HbA1c, TC, LDL, HDL, and TG values in the medical records as well as nutritional education, physical education, glucose self-monitoring, and patient satisfaction with the service. BMI was considered an indicator of metabolic control. The BDS (2012–2013) recommends the following targets for the metabolic control of diabetes patients:

1. HbA1c above the upper limit of the normal range, i.e., ≤7.0 % (53 mmol/mol), keeping in mind the goal can be individualized to the patient
2. sBP <130 mmHg for adults and <150 mmHg for elderly patients
3. dBP <80 mmHg for adults and <90 mmHg for elderly patients
4. BMI <25 kg/m^2^
5. Total cholesterol <5.2 mmol/L
6. HDL cholesterol >1.2 mmol/L for men and women
7. LDL cholesterol <2.6 mmol/L for adults without cardiovascular disease
8. Triglycerides <1.7 mmol/L.

Supporting process measurement indicators included treatments for diabetes (i.e., diet, oral hypoglycemic agent [OHA] monotherapy, OHA combination therapy, insulin monotherapy, and insulin plus OHA combination therapy), antihyperlipidemic agents, antihypertensive agents, and number of clinical visits (i.e., endocrinology service for diabetes care and others) in the last year. All health procedures and laboratory exams followed the same methodological protocol established in the hospital.

Statistical analyses were performed using SPSS for Windows version 20.0 (SPSS Inc., Chicago, IL, USA). For descriptive statistics, the normality and homoscedasticity of the distribution of all parameters were determined by the Kolmogorov–Smirnov test. Frequencies were accordingly expressed as means and standard deviations (SD) or medians and ranges where appropriate. The variation in sample distribution and mean differences were performed using the Mann–Whitney test.

The variables studied, were submitted to bivariate inferential analysis according to their clinical coherence by using the parametric Pearson test and nonparametric of Spearman test. Proportions were compared using the *χ*^2^ test or Fisher’s exact test where appropriate. The level of statistical significance was set at *p* < 0.05.

## Results

Data from a total of 1031 medical records were analyzed: there were 299 (29 %) T1DM patients (55.2 % female) and 732 (71 %) T2DM patients (68.0 % female). T2DM patients were significantly older than T1DM patients (*p* < 0.01). All patients received healthcare from the NBHCS at the same outpatient endocrinology service for diabetes care. No medical records contained information about patient satisfaction with their diabetes care. The treatment period for diabetes and number of clinical visits to the endocrinology service were greater in T1DM than T2DM patients (*p* < 0.01). However, T2DM patients required more “other” healthcare than T1DM patients (*p* < 0.01) (Table 1).

**Table 1:** Characteristics of type 1 and 2 diabetes mellitus patients

	*n*	T1DM + T2DM^a^	*n*	T1DM^a^	*n*	T2DM^a^	*p* value*
Age (years)	1030	57.0 (18–92)	298	31.0 (18–70)	732	62.0 (22–92)	<0.01
Weight (kg)	1015	74.0 (27.0–139.0)	281	66.3 (27.0–128.5)	706	77.4 (38.8–139.0)	<0.01
Height (cm)	988	160.0 (139.0–196.0)	281	164.0 (140.0–196.0)	706	1.59 (139.0–187.0)	<0.01
BMI (kg/m^2^)	987	28.1 (11.8–54.3)	281	24.3 (11.8–46,1)	706	29.8 (17.7–54.3)	<0.01
Number of clinical visits—DM (last year)	1030	2.0 (0–11)	298	3.0 (0–5)	732	2.0 (0–11)	<0.01
Number of clinical visit—other (last year)	1031	1.0 (0–57)	299	0.0 (0–37)	732	1.0 (0–57)	<0.01
Period of treatment for DM (years)	1027	7.0 (0–30.0)	297	9.0 (0–30.0)	730	6.0 (0–30.0)	<0.01

*T1DM* type 1 diabetes mellitus, *T2DM* type 2 diabetes mellitus, *BMI* body mass index

* Mann–Whitney *U* test

^a^Median (range)

The proportions of T1DM and T2DM patients who reached the goals for BMI differed significantly (Table 5). BMI was significantly higher in T2DM patients than T1DM patients (*p* < 0.01) (Table 1): 47.5 % (*n* = 142) of T1DM patients were overweight or obese compared to 85.1 % (*n* = 605) of T2DM patients (*p* = 0.001). Abdominal circumference was rarely measured in both T1DM and T2DM patients (1.7 % and 1.1 %, respectively). Regarding smoking status, 14 % (*n* = 42) of T1DM and 17.9 % (*n* = 131) of T2DM patients had data available; accordingly, 6.2 % (*n* = 23) and 5.6 % (*n* = 41) of T1DM and T2DM patients were smokers, respectively.

Most patients achieved metabolic control according to blood examination as a process measure in the last 6 months (93.69–99.42 %). The median values differed significantly between T1DM and T2DM patients: sBP, dBP, and TG were significantly higher in patients with T2DM (*p* < 0.01), whereas TC and LDL were significantly lower in patients with T2DM (*p* < 0.01). Furthermore, HDL was significantly higher in T1DM patients (*p* < 0.01) (Table 2). T1DM patients received fewer prescriptions for antihyperlipidemic agents, antihypertensive agents, and aspirin (*p* < 0.01) (Tables 3, 4).

**Table 2:** Process measurements and outcome measures of patients with type 1 and 2 diabetes mellitus

	*n*	T1DM + T2DM^a^	*n*	T1DM^a^	*n*	T2DM^a^	*p* value*
sBP (mmHg)	1025	120 (70–222)	296	120 (70–222)	729	130 (80–220)	0.00
dBP (mmHg)	1025	80 (40–130)	296	70 (40–121)	729	80 (51–130)	0.01
HbA1c (%)	1004	8.2 (4.1–16.2)	299	9.0 (4.1–16.2)	711	7.8 (4.3–16.0)	0.00
TC (mmol/L)	984	4.3 (1.9–9.1)	287	4.4 (2.6–8.3)	697	4.3 (1.9–9.1)	0.01
HDL (mmol/L)	986	1.1 (0.4–2.5)	288	1.2 (0.5–2.5)	698	1.0 (0.4–1.5)	0.00
LDL (mmol/L)	966	2.5 (0.4–6.8)	281	2.6 (1.0–6.7)	685	2.4 (0.4–6.8)	0.00
TG (mmol/L)	984	1.3 (0.2–9.3)	288	1.0 (0.3–7.9)	695	1.5 (0.2–9.3)	0.00

*T1DM* type 1 diabetes mellitus, *T2DM* type 2 diabetes mellitus, *dBP* diastolic blood pressure, *TC* total cholesterol, *HDL* high-density lipoprotein cholesterol, *LDL* low-density lipoprotein cholesterol, *TG* triglycerides, sBP systolic blood pressure

* Mann–Whitney *U* test

^a^Median (range)

**Table 3:** Frequencies of requests, prescriptions, and educational actions as process measures and outcomes indicators for patients with type 1 and 2 diabetes mellitus

	*n*	T1DM + T2DM (%)	*n*	T1DM (%)	*n*	T2DM (%)	*p* value
Foot examination	618	59.9	227	75.9	391	53.4	0.00*
Funduscopy	445	43.2	164	54.8	451	38.4	0.00*
Microalbuminuria	942	91.4	266	89.0	676	92.3	0,08*
Nutritional education	457	44.3	109	36.5	348	47.5	0.00*
Physical education	266	25.8	60	20.1	206	28.1	0.00*
Smoking cessation education	14	1.4	4	1.3	10	1.4	1.00**
Glucose self-monitoring	637	61.8	247	82.6	390	53.3	0.00*
ACE or ARA2 inhibitor prescription	677	65.66	123	41.13	554	75.7	0.00**
Aspirin prescription	525	50.9	60	20.1	465	63.5	0.00*

*T1DM* type 1 diabetes mellitus, *T2DM* type 2 diabetes mellitus, *ACE* angiotensin-converting enzyme

* *χ* ^2^ test

** Fisher’s exact test

**Table 4:** Supporting process measures of patients with type 1 and 2 diabetes mellitus

	*n*	T1DM + T2DM (%)	*n*	T1DM (%)	*n*	T2DM (%)	*p* value
Last prescription for DM							
Diet	16	1.5	0	0	16	2.1	0.00*
OHA monotherapy	145	14.0	0	0	145	19.8	
OHA combination therapy	120	11.7	0	0	120	16.3	
Insulin + OHA combination therapy	383	37.1	38	12.7	345	47.1	
Insulin monotherapy	367	35.7	257	86.0	110	15.0	
Antihyperlipidemic agents	738	71.6	132	44.1	606	82.8	0.00*
Antihypertensive agents	738	71.6	131	43.8	607	82.9	0.00**

*T1DM* type 1 diabetes mellitus, *T2DM* type 2 diabetes mellitus, *OHA* oral hypoglycemic agent, *ARA2* angiotensin II receptor antagonist

* *χ* ^2^ test

** Fisher’s exact test

T2DM patients underwent foot and eye examinations significantly less frequently than patients with T1DM (*p* < 0.01). However, the frequency of urine albumin screening was similar between groups (*p* > 0.05). Information regarding nutritional education was present in 44 % of the medical records (Table 3), but 98 % of the patients (*n* = 1008) did not receive nutritional consultation. Furthermore, physical education and mainly, education for smoking cessation were neglected by physicians in medical records as reflected by the low frequency of registration. Education for smoking cessation was similar between T1DM and T2DM patients (*p* > 0.05), whereas T2DM patients received physical and nutritional education significantly more frequently than T1DM patients (*p* < 0.01). Significantly more T1DM patients performed glucose self-monitoring than T2DM patients (*p* < 0.01) (Table 3).

HbA1c level was significantly lower in T2DM patients than T1DM patients (*p* < 0.01) (Table 2). Insulin plus OHA combination therapy and insulin monotherapy were the most frequently prescribed treatments. Insulin monotherapy was prescribed for 86 % of T1DM patients, and insulin plus OHA combination therapy was prescribed for 47 % of T2DM patients (Table 4).

The proportions of T1DM and T2DM patients who underwent clinical and laboratory evaluations, and met the BDS criteria differed significantly. T1DM patients reached most of the goals for metabolic control (Table 5). However, significantly fewer T1DM patients achieved the targets for HbA1c than T2DM patients (*p* < 0.01). Significantly more T1DM patients achieved the goals for sBP, dBP, HDL, and TG (*p* < 0.01). Meanwhile, significantly more patients with T2DM achieved the goals for TC and LDL (*p* < 0.05) (Table 5). Only 0.5 % (*n* = 5) of the patients reached all targets, and 1.1 % (*n* = 11) did not reach any.

**Table 5:** Numbers and proportions of type 1 and 2 diabetes mellitus patients who achieved the goals for metabolic control and screening for diabetic complications

	T1DM + T2DM	T1DM		T2DM		*p* value*
	*n*	*n*	Goals achieved % (*n)*	*n*	Goals achieved % (*n)*	
BMI	1010	299	52.5 (157)	711	14.9 (106)	0.00
sBP	1025	296	83.4 (247)	729	62.8 (458)	0.00
dBP	1025	296	88.8 (263)	729	82.0 (598)	0.00
HbA1c	1004	293	9.5 (28)	711	33.3 (237)	0.00
TC	984	287	76.3 (219)	697	81.1 (565)	0.09
HDL	986	288	55.6 (160)	698	29.7 (207)	0.00
LDL	966	281	46.2 (130)	685	58.2 (399)	0.00
TG	984	288	84.0 (242)	696	58.0 (404)	0.00

*T1DM* type 1 diabetes mellitus, *T2DM* type 2 diabetes mellitus, *BMI* body mass index, *sBP* systolic blood pressure, *dBP* diastolic blood pressure, *TC* total cholesterol, *HDL* high-density lipoprotein cholesterol, *LDL* low-density lipoprotein cholesterol, *TG* triglycerides

* *χ* ^2^ test

## Discussion

In the present study, all outpatients received healthcare and treatment by an endocrinologist at a public secondary care service part of the NBHCS. The proportions of patients who achieved the targets of process measures, outcomes indicators, and supporting process measures varied substantially, and there were significant differences between T1DM and T2DM patients.

Almost no patients achieved all targets or failed to achieve any. Another study of T1DM patients in Brazil reports similar results [14]. Between 0.2 % and 7.0 % of patients with T2DM who had a disease duration near 10 years achieved at least three or all targets, while between 8.8 and 11.4 % did not achieve any [17–19]. In contrast, one epidemiologic American study recent, large-sized, suggests that 33.4–48.7 % of persons with diabetes still did not meet the targets for glycemic control, blood pressure, or LDL cholesterol level and only 14.3 % met the targets for all three of these measures and for tobacco use [20]. These data are different to those found in Brazil and indicate the need for changes in care plan and national policies for diabetes management.

The present results reaffirm that the achievement of therapeutic goals for patients with T1DM or T2DM is a complicated issue. Difficulty achieving appropriate glycemic control, blood pressure, and lipid levels is associated with health service availability, social and economic factors, and education levels [17–19]. Clinical inertia and an inadequate support network can result in patients failing to initiate or intensify treatment in a timely manner. It should be noted that adherence to treatment is an issue with significant impact on the obtainment of goals, but was not assessed in our study, as the retrospective character of the collection and the absence of records of this parameter in the patient [21–23].

Although most patients had T2DM in the present study, the frequency of T1DM was relatively high, probably because the study was conducted at a secondary healthcare service. Patients with T1DM rarely receive treatment at primary care centers in Brazil [14]. However, primary care as part of the NBHCS must be assured to all T1DM patients [12]. T2DM patients had more visits to secondary care services other than endocrinology, due to presence of the others comorbidities, justified by the older age and high BMI. As expected, T1DM patients made more clinical visits to an endocrinologist; this is because of the greater complexity of basal treatment, which usually requires multiple administrations of insulin and the need for frequent monitoring [25–27].

As expected, patients with T2DM were significantly older and had significantly more chronically diseases and diabetes-related complications. It should be noted that the comparison of outcomes between T1DM and T2DM patients is hampered by the older age of T2DM patients and much longer disease duration of T1DM patients [28].

Almost half of the patients in the present study with T1DM were overweight. However, even more patients with T2DM were overweight in accordance with the tendency of obesity as a global epidemic. In 2014, 39 % of the global adult population was overweight [29]. The proportions of Brazilian patients with T1DM and T2DM who are overweight are reported to be 29.4 and 75.4 %, respectively [14, 19], which are less than those in the present study. In the United States of America, 87.7 % of outpatients with T2DM have a BMI exceeding 25 kg/m^2^ [17], which is similar the results of the present study.

Overweight/obesity is a major risk factor for non-communicable diseases and can lead to metabolic complications [29]. Almost all patients in the present study were screened for metabolic control. However, examinations to detect diabetes-related complications were rarely requested, similar to results found in other Brazilian populations with T1DM and T2DM [14, 19]. Even though healthcare providers from community health centers in the United States of America consider process measures extremely important, 25 % report forgetting to request funduscopy and foot examinations [31], which is a lower proportion than that in the present study. In an Italian study of 114,249 outpatients with T2DM, foot examinations were ordered for 22.4 % and funduscopy for 48.1 % during the preceding year, respectively [18]. Assessment of microalbuminuria was performed for 92.3 % of the patients with T2DM in the present study.

Eye and foot examinations were performed more frequently for patients with T1DM, which may be associated with the fact that 90.5 % of T1DM patients did not achieve the target for HbA1c; these findings are concordant with those of other studies on T1DM patients [14, 17, 18, 33]. In a Brazilian multicenter study involving public secondary and tertiary clinics of the NBHCS that included 1800 T1DM patients (mean age 30.3 ± 9.7 years old), 88.4 % achieved HbA1c ≥7 % [14]. In the present study, 33 % of T2DM patients achieved the HbA1c target. The proportion of T2DM patients with similar age and duration of diabetes who achieved HbA1c <7 % ranges between 37 and 46 % in observational studies worldwide [14, 17, 18, 33]. These results are worrisome and indicate many patients with T2DM have poor glycemic control, as HbA1c is the best predictor of microvascular and macrovascular outcomes in such patients [30, 34].

Despite glucose self-monitoring and insulin monotherapy or insulin plus OHA combination therapy, T1DM patients had significantly higher HbA1c than T2DM patients. Adults with T2DM had HbA1c values similar to those in others studies involving patients with similar age and duration of diabetes (between 7.4 ± 1.5 and 7.9 ± 1.8 %) [17, 18, 35].

Only approximately half of the total patients performed glucose self-monitoring. Healthcare providers report teaching patients to perform home glucose monitoring is too time consuming and that some patients cannot afford to do so [27]. Others reasons include a lack of appropriate equipment because of high costs to the NBHCS. Very long-term intensive glycemic control improves cardiovascular outcomes [14]; glucose self-monitoring is important considering most patients with T1DM and T2DM have multiple cardiovascular risk factors [14, 36] and that it could help achieve the goal for HBA1c.

The latest prescription for diabetes treatment differed between T1DM and T2DM. All patients with T1DM received insulin or insulin plus OHA, whereas the most frequent prescription for patients with T2DM was insulin plus OHA therapy, which is corroborated by several observational studies worldwide [14, 36]. The frequencies of treatment with diet alone, a single OHA, and insulin monotherapy were lower in the present study than another Brazilian study of patients with T2DM [19].

Besides HbA1c, cardiovascular risk is related to central obesity, hypertension, and dyslipidemia in T1DM and T2DM [28, 36, 37]. However, abdominal circumference was not measured in most patients in the present study.

Approximately 24 and 20 % of Brazilians have hypertension and dyslipidemia, respectively [12]. In the present study, patients with T2DM had higher blood pressure and TG levels, which is consistent with previous studies reporting increased risks of hypertension and dyslipidemia in patients with diabetes [38–41]. On the other hand, TC, HDL, and LDL levels were significantly higher in T1DM patients, whereas the number of antihyperlipidemic prescriptions was lower.

The present results showed 28.5 and 19.3 % of T2DM patients achieved the goals of sBP and dBP, respectively; thus, the present patients show better blood pressure control than those in another Brazilian study [19]. In a study in the United States of America, 58.9 % of T2DM patients showed good blood pressure control [35]. The median sBP in the present study is lower than the mean value (141 ± 19 mmHg) in Italian outpatients with T2DM [18]. Poor sBP and dBP are frequently observed in patients with T2DM [42].

As blood pressure is elevated in T2DM, the prescriptions of antihypertensive agents, ACE inhibitors, and ARA2 were more frequent in T2DM patients than T1DM patients in the present study. The frequencies of prescriptions of antihypertensives, ACE inhibitor, angiotensin receptor blockers, and aspirin in the present study are better than those in American and Italian studies of T2DM outpatients [17, 18].

In the present study, T1DM patients had significantly higher cholesterol fractions; 76.3 and 84.0 % of T1DM patients achieved the TC and TG goals, respectively, and 44.1 % were receiving antihyperlipidemic agents.

Patients with T2DM in the present study achieved the goals for TC, TG, and LDL more frequently than those in another Brazilian study of T2DM patients with similar age and duration of diabetes [19]. Studies from the United States of America and Italy report similar results: 43.7 and 29.8 % of the outpatients with T2DM had LDL <100 mg/dL, respectively [17, 18]. However, in a recent American study, 62.8 % of T2DM patients achieved the target for LDL [35]. In the present study, fewer patients with T1DM were prescribed antihyperlipidemic agents than T2DM patients. These results are better than those reported in the United States of America (58.8 %) [17].

The greater proportions of antihyperlipidemic, antihypertensive, and aspirin prescriptions as well as the frequent insulin recommendation for T2DM treatment suggest a change in clinical inertia and earlier treatment intensification. However, these results do not directly demonstrate the adequacy of a support network with multidisciplinary diabetes care teams focusing on diabetes self-management education as well as social, economic, and environmental improvements in order to increase treatment adherence (mainly physical activity and diet recommendations).

The present results show nutritional and physical education activities are frequently omitted from most medical records. Nutritional education was recorded in less than half of the total patients’ records; it was most frequently recorded for T2DM patients and was probably prescribed by physicians, because only 2 % of patients had a nutritional consultation recorded in their medical records.

The present results suggest physicians do not appropriately refer patients to dietitians, probably because of the lack of dieticians in the healthcare system or because physicians considered their dietary recommendations sufficient. Providers state patients are unable to follow a diet or exercise program regularly but consider special diets and exercise very or extremely important processes; therefore, dietitians are one of the most common health professionals to whom primary care clinicians refer their patients [17, 31]. A regular and balanced diet in conjunction with pharmacotherapy is fundamental for the treatment of diabetes [34, 43, 44].

Moreover, smoking cessation was often neglected by physicians according to the medical records. Smoking status was registered in few medical records, indicating an assumption of a reduced number of smokers. The proportion of T2DM patients whose smoking status was recorded in the present study is lower than that in a multicenter study in Brazil (54.5 %) [19]. Meanwhile, the frequency of current smoking status in T1DM patients is similar to that in another Brazilian multicenter (7.3 %) [14].

Trained care physicians and multidisciplinary diabetes care teams can aid diabetes self-management education, blood pressure control, lipid profile, early improvement intensive glycemic control, insulin and hypoglycemic agent dose adjustments, and hyperglycemia treatment, all of which slow the progression of chronic complications and improve quality of life [28, 41–43].

Social and environmental burdens are the main reasons for necessary lifestyle modifications in the management of diabetes. Nonadherence to self-care behaviors is common among patients with diabetes and is often associated with a poor understanding of the disease and its treatment [24].

The management of outpatients with T1DM and T2DM, including improving metabolic control and reducing the risk of developing diabetes-related complications, is challenging for healthcare systems. A large integrated and redesigned healthcare system with more healthcare professionals and trained multidisciplinary teams, patient education programs, prioritized public policies on non-communicable diseases, and improved structural and financial access to care are necessary to promote appropriate interventions to spur behavioral changes in such patients [31, 32].

Patient satisfaction with diabetes care is a component of quality measurement, but was not found in any medical record in the present study. The quality of diabetes care, which includes process and intermediate outcome indicators, must be addressed more carefully in order to promote continuous improvement initiatives and develop a more effective healthcare model for these patients [14, 18, 19].

A limitation of the study is that it was conducted in a single service, so the generalization of data should be performed with caution. However, this limitation may have been softened, due to high sample number and the wide variability of patients assessed, considering that the service in question is an important reference center for the whole region.

## Conclusion

The proportions of patients with T1DM and T2DM who achieve the goals of good clinical practice during routine secondary endocrine care in the NBHCS Support Network vary with respect to the parameters evaluated. Almost no patients achieved all targets. Many patients are overweight and have not achieved the targets for HbA1c, lipid profile, or blood pressure control. Furthermore, nutrition, physical activity, and smoking cessation education activities are neglected in medical records, and examinations for detecting diabetes complications are not sufficiently requested.

There are limitations when comparing patients with T1DM and T2DM because of the differences in the disease characteristics; nevertheless, the therapeutic targets are the same for both diseases. Moreover, the achievement of goals is indicative of the healthcare quality of the Support Network, suggesting care practice must be reviewed to improve the metabolic control of patients with diabetes. Therefore, further prospective studies investigating healthcare quality through the assessment of care for chronic conditions by using direct questionnaires and measurement indexes are warranted.
